# Multimodal Interventions Targeting Gut Microbiota and Microbial Metabolites in Cognitive Impairment

**DOI:** 10.7759/cureus.85688

**Published:** 2025-06-10

**Authors:** Paulina Horwat, Agnieszka Mariowska, Anita Szymanska, Marta Dzieciatkowska, Weronika Pierudzka

**Affiliations:** 1 Hospital Medicine, University Clinical Hospital in Poznan, Poznań, POL; 2 Department of Cardiology, Poznan University of Medical Sciences, Poznań, POL; 3 Hospital Medicine, University Clinical Hospital in Poznan, Poznan, POL

**Keywords:** gut-brain axis, gut microbiome, microbiome-derived metabolites, mild cognitive impairment (mci), multimodal intervention

## Abstract

Mild cognitive impairment (MCI) is a transitional stage between normal aging and Alzheimer’s disease (AD). Recent studies suggest that alterations in gut microbiota and microbial metabolites are associated with cognitive decline, highlighting the gut-brain axis as a potential therapeutic target. This narrative review explores current evidence on the relationship between gut microbiota, microbial metabolites, and MCI. It summarizes intervention strategies including probiotics, prebiotics, synbiotic, fecal microbiota transplantation, dietary modifications, medicinal herbs, phytochemicals, metformin, and lifestyle factors. Probiotic strains such as *Lactobacillus* and *Bifidobacterium* have shown cognitive benefits. Adherence to the Mediterranean and MIND diets, as well as metformin use, is associated with lower MCI risk. Novel strategies, including plant-based compounds and nature exposure, show promise in modulating gut microbiota and improving cognitive outcomes. Gut microbiota modulation represents a promising avenue for early intervention in MCI. Personalized, multifactorial approaches based on individual microbiome profiles may enhance prevention and management strategies. However, more high-quality clinical trials are needed to establish evidence-based guidelines.

## Introduction and background

Introduction

Cognitive impairment is characterized by a reduction in cognitive abilities that surpasses typical age-related changes, yet does not fulfill the criteria for dementia. It encompasses deficits in various domains such as attention, memory, executive function, and visuospatial abilities [1,2]. This condition ranges from mild cognitive impairment (MCI) to more severe forms that may progress to dementia [1,3]. The prevalence of cognitive impairment varies depending on the diagnostic criteria used and the study population. In young-old adults (60-64 years), the prevalence rates range from 1.5% to 23.5%, based on different diagnostic concepts [4]. In a longitudinal study of community-dwelling young-old subjects, the prevalence of aging-associated cognitive decline increased from 13.4% at baseline to 23.6% after four years [5]. Additionally, cognitive impairment is common in specific populations, such as 30% of stroke survivors [6], and a significant proportion of patients with heart failure [2]. Cognitive impairment has become a modern societal issue owing to its substantial public health implications. It is associated with an increased risk of adverse medical, psychiatric, and cognitive outcomes, including progression to dementia [7,8]. This condition places a significant burden on individuals, caregivers, and healthcare systems, leading to higher rates of disability, impaired self-care, increased healthcare costs, and higher mortality rates [2].

Mild cognitive impairment (MCI) represents a critical window for intervention, as it is often regarded as a potentially reversible stage preceding Alzheimer’s disease (AD) [1]. In recent years, increasing attention has turned to the gut-brain axis as a novel and biologically plausible contributor to cognitive impairment. Specifically, alterations in gut microbiota composition and their derived metabolites have been associated with the pathogenesis and progression of cognitive decline [9,10]. Emerging research suggests that individuals with MCI exhibit distinct gut microbial profiles compared to healthy individuals. For instance, a reduction in the abundance of genera such as Ruminococcus, Butyricimonas, and Oxalobacter, alongside an increase in Flavonifractor, has been observed in those with MCI [9]. Similarly, other studies have identified increased levels of bacterial taxa such as Erysipelatoclostridiaceae, Erysipelotrichales, Patescibacteria, Saccharimonadales, and Saccharimonadia in MCI patients [10]. These microbial changes are not merely incidental; they have been correlated with clinical symptoms and may play a functional role in neurodegenerative processes [9,10]. In parallel, key microbial metabolites - including short-chain fatty acids (SCFAs), tryptophan catabolites, trimethylamine N-oxide (TMAO), and secondary bile acids - are increasingly recognized for their capacity to cross the intestinal barrier and influence central nervous system activity [11,12]. In MCI and AD, a progressive decline in SCFAs and disruptions in tryptophan metabolism have been reported, implicating these metabolites in neuroinflammation, blood-brain barrier integrity, and synaptic function [13,14]. These findings underscore the relevance of the gut microbiota as both a marker and modifiable factor in early cognitive impairment. Consequently, interventions aimed at restoring microbial balance and enhancing the production of beneficial metabolites hold promise for preventing or delaying cognitive decline.

Based on recent research, we conducted a review to assess the clinical relevance of multimodal approaches to cognitive impairment treatment, summarizing current perspectives for healthcare practitioners. As a novel aspect, we discuss the implications of metformin use and nature contact in the context of cognitive decline. Both interventions have demonstrated potential cognitive benefits, with their proposed mechanisms of action involving modulation of the gut microbiota composition, suggesting that macrobiotic shifts may play a central role in mediating these effects. Although the exact mechanisms linking gut microbiota and its metabolites to cognitive function remain largely preclinical, several evidence-based lifestyle interventions can already be recommended in clinical practice.

Methods

Study Design

To ensure the objectivity of this review, a comprehensive literature search was conducted. The PubMed database was queried using the keywords: (‘gut microbiome’ OR ‘microbiome metabolites’) AND ‘cognitive decline’. Inclusion criteria were as follows: original research articles, reviews, scoping reviews, systematic reviews, or meta-analyses; studies involving human or animal models; publications examining the relationship between the gut microbiome or its metabolites and cognitive impairment (including MCI and AD); published in English from 2020 onward. Exclusion criteria included: case reports, articles published prior to 2020, non-English publications, and studies focusing solely on age-related cognitive decline without specific mention of microbiota-related mechanisms. Additionally, reference lists of selected articles were manually screened to identify supplementary studies relevant to the review. A total of 347 articles were initially identified. Only data providing novel insights relevant to the review objective were extracted and synthesized to support the conclusions presented. No statistical analysis was performed.

Risk of Bias

This narrative review includes a broad spectrum of study designs, encompassing narrative reviews, observational studies, experimental animal research, clinical trials (including randomized controlled trials), and systematic reviews. Given this heterogeneity, the risk of bias across included studies varies considerably. Several older studies and narrative reviews lack standardized methods for assessing cognitive outcomes or controlling for confounders, thereby limiting their internal validity. Some of the recent experimental and clinical studies, particularly those examining the gut-brain axis and interventions like probiotics or fecal microbiota transplantation, utilized randomized controlled designs and reported standardized outcome measures, which strengthens the evidence base; however, small sample sizes and limited follow-up duration may reduce their generalizability. Observational studies included in the review may be subject to selection bias, residual confounding, and reverse causation. Additionally, the inclusion of animal studies introduces concerns regarding translational applicability to human populations. Overall, while many studies contribute valuable insights into the multifactorial nature of cognitive impairment and its potential modulation through gut microbiota and diet, the varied methodological quality necessitates cautious interpretation of findings and underscores the need for more rigorous, longitudinal, and standardized human trials.

Description of the state of knowledge

Gut - Brain Axis

The gut-brain axis (GBA) is an intricate system facilitating two-way communication between the gut microbiota and the central nervous system. This axis involves various pathways, including the immune system, enteric nervous system, vagus nerve, and microbial metabolites [15,16]. The gut microbiota produces a wide range of bioactive compounds that can cross the blood-brain barrier (BBB) or significantly affect brain function, playing a crucial role in the microbiota-gut-brain axis [17]. Additionally, in patients with cognitive impairment, disruption of tight junction integrity has been associated with increased intestinal permeability, or “leaky gut,” allowing microbial metabolites like lipopolysaccharides (LPS) to enter systemic circulation and trigger neuroinflammation [18]. Microbial metabolites, such as SCFAs, trimethylamine N-oxide (TMAO), tryptophan derivatives, and bile acids, are key players in this communication system [12,17,19]. SCFAs, produced through the fermentation of dietary fiber, serve as energy substrates for intestinal epithelial cells, influence host epigenetic, activate G protein-coupled receptors, and inhibit pathogenic microbial infections [19]. TMAO and secondary bile acids are associated with cognitive function and neurodegenerative disorders [12]. Dysbiosis, or an imbalance in gut microbiota composition, has been linked to neuroinflammation and various neurological conditions [20]. In individuals with subjective cognitive decline, a reduction in the anti-inflammatory genus *Faecalibacterium *was noted, alongside an increased presence of *Prevotella *and *Dehalobacterium*, which correlated with lower scores in cognitive function. Elevated levels of Aeromonas and lipopolysaccharide (LPS) were noted in a murine model of hippocampal neuronal apoptosis and spatial memory impairments [21]. Stress conditions can impair communication across the BBB, allowing gut microbiota to affect brain function [16]. The influence of the gut microbiome on cognitive disorders, including AD, has been observed, with probiotics showing potential in managing cognitive disorders [22] or as a potential early biomarker for the detection and further monitoring of developing AD [23]. There is great interest in understanding inflammatory responses in the central nervous system (CNS) [15]. The induction of inflammation in neurodegenerative disorders can activate neurodegenerative processes. In the brain, immune responses are carried out by microglia, astrocytes, and mast cells [15]. The gut-brain connection involves the microbiota’s role in producing neurotransmitters and fatty acids. Alterations in neurotransmitter levels affect neuronal communication [24]. The CNS reacts by adjusting the functions of the autonomic nervous system and the activity of the hypothalamic-pituitary-adrenal axis. Gut microbiota influences neural function by affecting glial and mast cells, while also affecting neurone-microglia relationships and synaptic changes [15].

Microbial Metabolites Relevant to Cognitive Function

Table 1 outlines the principal microbial metabolites implicated in the microbiota-gut-brain axis (MGBA) and their respective roles in modulating cognitive processes. SCFAs, including butyrate, acetate, and propionate, are generated through microbial fermentation of dietary fiber and exert neuroprotective effects by modulating neuroinflammation, enhancing BBB integrity, influencing host epigenetic regulation, and activating G protein-coupled receptors (GPCRs) [19,25,26,27,28,29]. These metabolites also serve as energy substrates for intestinal epithelial cells and contribute to the maintenance of systemic immune homeostasis [19]. Conversely, elevated levels of trimethylamine-N-oxide (TMAO), a metabolite produced by microbial metabolism of dietary choline and L-carnitine, are associated with vascular dysfunction, mitochondrial stress, BBB disruption, and tau and Aβ pathology, thereby contributing to cognitive decline and AD progression [12,27,30]. Although some evidence from Mendelian randomization studies suggest no causal role of microbiome-derived metabolites in AD pathology, this remains a subject of ongoing investigation [31]. The aforementioned study relies heavily on correlational data, which limits causal inference and poses a risk of overinterpretation. Additionally, the methodologies used to assess gut microbiota vary significantly across studies, potentially leading to inconsistent findings. Moreover, there is a notable scarcity of large-scale, randomized controlled trials in humans.

**Table 1:** Main gut microbiome metabolites in the gut-brain axis

Metabolite	Main Sources	Key Functions	Cognitive/Neurological Impact	References
Short-chain fatty acids (SCFAs) (e.g., butyrate, acetate, propionate)	Fermentation of dietary fiber by gut microbiota	- Cross the blood-brain barrier (BBB) and act directly on brain tissue, serve as energy substrates for intestinal epithelial cells, regulate immune responses and systemic inflammation, activate G protein-coupled receptors (GPCRs), influence host epigenetics and energy metabolism, prevent pathogenic microbial infections	- Exhibit neuroprotective and anti-inflammatory effects, mitigate neuroinflammation and chronic cerebral hypoperfusion, modulate neuroendocrine function and synaptic plasticity	[12, 19, 25, 26, 28, 29]
Trimethylamine-N-oxide (TMAO)	Produced from dietary choline and L-carnitine by gut microbiota; converted in liver	- Alters vascular function, crosses and disrupts the BBB, induces mitochondrial stress, impairs synaptic plasticity	- Associated with cognitive decline and neurodegenerative diseases (e.g., Alzheimer’s disease), contributes to tau and Aβ pathology, contradictory evidence from Mendelian randomization studies	[12, 27, 30, 31]
Tryptophan metabolites (e.g., kynurenic acid, quinolinic acid, serotonin, indole derivatives)	Tryptophan catabolism via the kynurenine pathway (KP); modulated by gut microbiota	- Modulate central nervous system (CNS) inflammation, influence serotonin and indole production, regulate immune responses, modulate neurotransmitter levels and neural activity	- Imbalance between neurotoxic (e.g., QUIN) and neuroprotective (e.g., KYNA) metabolites linked to cognitive and psychiatric disorders, affects behavior and cognition, potential targets in conditions like autism, depression, and AD	[32-35, 37, 38]
Gamma-aminobutyric acid (GABA)	Synthesized by gut microbes (e.g., *Blautia *spp.) via microbial enzymes	- Major inhibitory neurotransmitter in the CNS, maintains excitatory/inhibitory balance, influenced by microbial and tryptophan metabolism	- Critical for maintaining normal brain function, altered GABA levels implicated in anxiety, depression, and cognitive decline	[15, 33, 34, 36]

Tryptophan metabolism, primarily via the kynurenine pathway (KP), yields neuroactive metabolites such as kynurenic acid (KYNA) and quinolinic acid (QUIN), which exert neuroprotective or neurotoxic effects, respectively. Dysregulation of this pathway has been implicated in the pathogenesis of several neuropsychiatric and neurodegenerative disorders [32,33]. Notably, probiotic interventions have been shown to modulate KP activity and mitigate behavioral impairments in preclinical models [34]. Gut microbiota modulates this pathway both directly and indirectly, influencing the balance of KP metabolites, as well as the synthesis of serotonin and indole derivatives, which are essential for maintaining neurochemical homeostasis [32,35].

Gamma-aminobutyric acid (GABA), the primary inhibitory neurotransmitter in the CNS, is also influenced by the gut microbiome. Specific microbial taxa, such as *Blautia *spp., contribute to GABA production via enzymes like putrescine aminotransferase, thereby influencing excitatory/inhibitory neurotransmission dynamics [15,33,34,36]. These findings underscore the complex interplay between microbial metabolites and central neural pathways, offering promising targets for therapeutic modulation of cognitive impairment.

## Review

Intervention strategies targeting the gut microbiota and metabolites

Probiotics

Probiotics have demonstrated promising outcomes in enhancing cognitive function in individuals with cognitive impairment, particularly those diagnosed with AD or MCI. Multiple studies highlight that probiotics can improve memory deficits, reduce synaptic and neuronal damage, and decrease microglial activation in animal models of cognitive decline [39]. Human trials involving AD, MCI, and healthy older adults have reported cognitive improvements-such as enhanced attention, recall, visuospatial, and executive functions-after 12 to 24 weeks of probiotic supplementation, as measured by Mini Mental State Examination (MMSE) and Montreal Cognitive Assessment (MoCA) scores [40,41]. However, efficacy may vary with disease severity; for example, patients with severe AD did not show significant cognitive improvement in one study [40]. A recent meta-analysis also questioned the broad effectiveness of probiotics by reporting no significant overall cognitive benefit, precisely in the MMSE (SMD: 0.28, 95%CI −0.35-0.91, p = 0.38) and MoCA scores (SMD: 0.51, 95%CI −0.49-1.52, p = 0.33) [42].

Mechanistically, probiotics exert their effects by modulating the gut microbiota composition, promoting beneficial bacteria while inhibiting pathogenic species, which leads to reduced gut permeability and systemic and neuroinflammation. They preserve BBB integrity and modulate immune responses by enhancing anti-inflammatory cytokine production and inhibiting bacteria-related signaling pathways such as caspase-11/caspase-1 and α-kinase 1 (ALPK1) expression in both gut and brain tissues [39,43,44].

**Figure 1:**
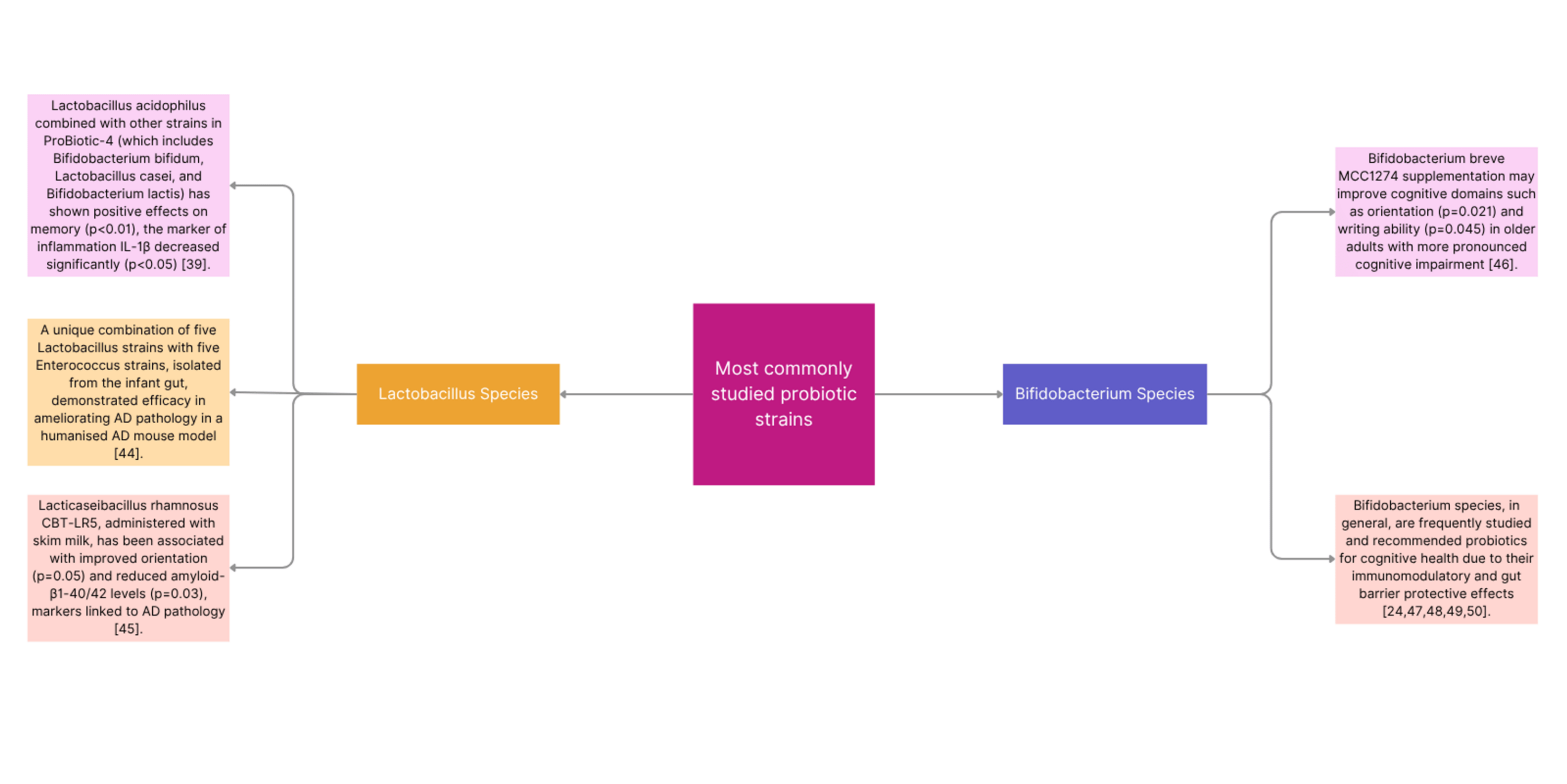
Summarization of most commonly studied probiotic strains

The characteristics and most commonly used probiotics strains are summarized on the Figure 1. Research focuses mainly on the *Lactobacillus *strains [39,44,45] and *Bifidobacterium *strains [24,46-50].

Although probiotics present potential as adjunct therapies for cognitive decline, the variability in study design, strain selection, and intervention duration limits firm conclusions. Notably, probiotic supplementation does not always lead to significant changes in overall gut microbiota composition, suggesting that beneficial effects might be mediated by specific microbial metabolites rather than broad microbiome shifts [46]. Therefore, future research should focus on standardizing probiotic formulations and further elucidating the molecular mechanisms-particularly metabolite-mediated pathways-underlying the GBA in cognitive function.

Prebiotics and Synbiotics

Prebiotics and synbiotics have demonstrated potential for managing cognitive impairment, although further investigation is required to establish definitive guidelines. Importantly, the influence of prebiotics and synbiotics on cognitive abilities seems to be linked to their impact on gut ecology and the production of SCFAs [49]. A study examining DSS-induced cognitive dysfunction in mice revealed that synbiotic supplementation with *Corni fructus* (as a prebiotic) and *Limosilactobacillus reuteri* (as a probiotic) significantly enhanced cognitive function, as assessed using the Y-maze and passive avoidance tests [51]. This synbiotic formulation aids in memory restoration by boosting the cholinergic system and diminishing tau and amyloid β pathologies [49, 51]. The aforementioned study found that synbiotics ameliorated dysbiosis by modulating the gut microbiota and increasing SCFA concentrations in the feces [51]. Nonetheless, it is important to recognize that the impact of prebiotics and synbiotics can differ based on the particular formulation and the unique gut environment of an individual. For instance, certain research has indicated changes in the microbial community without notable enhancements in disease outcomes or biochemical indicators [52]. Although specific product recommendations for patients are limited based on the information provided, some prebiotics that have shown potential include inulin and fructooligosaccharides [53]. Furthermore, combinations of *Lactobacillus* and *Bifidobacterium* strains with these prebiotics have been tested in human clinical trials [52]. However, the concept of synergistic synbiotics, which combine elements that can effectively improve health even in non-responders, may offer a more promising approach for future interventions [52]. Moreover, the positive effects of prebiotics are mainly observed in animal studies [47]. To identify the most effective prebiotic and synbiotic formulations for addressing cognitive impairment, further investigation, especially through high-quality clinical trials, is essential.

Fecal Microbiota Transplant

Fecal microbiota transplantation (FMT) has demonstrated potential in reducing the symptoms of cognitive impairment, though further studies are necessary to confirm its effectiveness. Research has indicated that FMT can lead to enhancements in cognitive abilities in individuals with MCI and dementia. A preliminary study indicated that FMT might preserve and enhance cognitive abilities in individuals with MCI by modifying the composition of gut microbiota and influencing serum metabolomics [54]. Another study observed significant improvements in clinical symptoms and cognitive function in patients with dementia receiving FMT compared with the control group [55]. The mechanisms underlying FMT’s effects of FMT on cognition have not yet been fully elucidated. FMT appears to modulate the gut microbiota composition, reduce inflammation, and influence glucose metabolism in the brain. Research has indicated that FMT can modify the bacterial taxa and diversity in recipient mice, influencing both brain glucose uptake and cognitive abilities [56]. In a separate study involving mouse models of AD, FMT was shown to enhance cognitive function and decrease inflammatory markers by modulating the TLR4/MyD88/NF-κB signaling pathway [57]. While these results are encouraging, it is important to note that FMT for cognitive impairment remains experimental. Current indications for FMT primarily focus on recurrent *Clostridioides *difficile infection [58]. To confirm the clinical effectiveness and safety of FMT for treating cognitive disorders, more extensive controlled trials are necessary to develop standardized guidelines. As research progresses, FMT may emerge as a potential therapeutic option for cognitive impairment, particularly in cases in which traditional treatments have been ineffective, however for the present date it remains as the experimental method.

Diet-Based Interventions

Diet-based interventions have shown promising results in the management of cognitive impairments. Numerous research studies have shown that certain dietary habits can effectively ease symptoms and lower the risk of cognitive decline [59]. The Mediterranean diet (MeDi) is frequently linked to a reduced likelihood of developing MCI and AD. A systematic review and meta-analysis revealed that individuals who closely follow the MeDi diet experience a 25% decrease in the risk of MCI (RR=0.75; 95% CI: 0.66-0.86; p=0.002) and a 29% reduction in the risk of AD (RR=0.71; 95% CI:0.56-0.89; p=0.063) [59]. Similarly, the MIND (Mediterranean-DASH Intervention for Neurodegenerative Delay) diet, which integrates aspects of both the Mediterranean and DASH diets, has been associated with a reduced risk of MCI (OR=0.23; 95% CI=0.06~0.99; p=0.04)[60]. The combination of Mediterranean and ketogenic diets has been demonstrated to enhance fecal butyrate production, which is inversely correlated with AD biomarkers [24,45]. Diets that are plant-based and high in fiber and polyphenols have been found to encourage gut bacteria to produce beneficial SCFAs, which possess anti-inflammatory properties and may enhance cognitive function [61]. Conversely, unhealthy plant-based diets high in refined grains and added sugars are associated with cognitive impairment [62]. Specific dietary components have also been identified as potentially beneficial for cognitive health. Whole grains, nuts, and eggs consumed once a week or more are associated with a lower risk of MCI (95% confidence interval 1.35, 3.60) [62]. Additionally, krill oil, which is rich in DHA/EPA and astaxanthin, has shown promise in improving cognitive function and reducing amyloid β concentrations in early cognitive impairment [63]. In the scientific evidence available suggests that patients with cognitive impairment may benefit from diets such as the Mediterranean diet, MIND diet, and plant-based diets that are high in whole grains, nuts, and omega-3 fatty acids. These dietary approaches have been found to support gut health, decrease inflammation, and slow down cognitive decline [27,64]. Nonetheless, it is crucial to acknowledge that further long-term randomized controlled trials are necessary to bolster the evidence and to create personalized dietary strategies for cognitive health.

Medical Plants and Phytochemicals

Medicinal plants and phytochemicals hold potential in mitigating cognitive decline by affecting the composition of gut microbiota and the production of metabolites. Aloe polysaccharides (APs) have demonstrated notable enhancements in cognitive behavioral issues induced by high-fat diets [65]. APs alter the gut microbiota by boosting the presence of beneficial bacteria like Akkermansia and decreasing harmful bacteria such as Helicobacter, which enhances the integrity of the intestinal barrier and reduces inflammation in both the brain and jejunum [65]. Although APs show positive effects, the impact of other phytochemicals on cognitive function varies. Anthocyanins and polyphenols enhance cognition, reduce inflammation, and influence the balance between SCFAs and TMAO balance [66]. Lignans have opposite effects on *Akkermansia muciniphila* levels. Intervention effectiveness depends on the baseline gut microbiota and polyphenol degraders [66]. *Polygonatum sibiricum* polysaccharide (PSP) acts as a prebiotic, promoting beneficial bacteria that produce SCFAs and maintain intestinal barrier integrity. PSP mitigates cognitive impairment in AD by boosting the population of beneficial bacteria and decreasing the accumulation of intestinal Aβ. It modulates immune cell balance, improves barrier function, and reduces neuroinflammation, potentially enhancing cognitive function in neurological disorders [67]. Already thirty medicinal plants that affect mental health disorders showed potential gut microbiota interactions. Plants containing polyphenols and polysaccharides have shown positive effects under dysbiotic conditions [68]. Although medicinal plants show promise for cognitive impairment, no single agent suits all individuals owing to gut microbiota variability [66].

Metformin

Metformin has been shown to influence gut microbiota composition and its metabolites, potentially leading to improvements in cognitive function [69]. This study demonstrated that metformin alters gut microbiota, which is necessary for protection against age-related cognitive decline in mice. Specifically, metformin enriched the abundance of Akkermansia muciniphila, a beneficial gut bacterium [69]. A. muciniphila administration enhanced cognitive abilities in older mice, primarily novel object recognition (p<0.01) by influencing inflammation-related pathways and lowering the levels of the pro-inflammatory cytokine interleukin-6 (IL-6) in both peripheral blood and hippocampal profiles [69]. This indicates that the cognitive advantages of metformin might be partially due to its impact on the gut microbiome. Additionally, metformin's influence on the gut microbiome could interact with other metabolic pathways that are important for cognitive function, for example TMAO and bile acid metabolism [12,70,71,72]. While these studies provide compelling evidence for the potential of metformin to improve cognitive function through microbiome modulation, it is important to note that these studies did not specify a particular dosage for cognitive benefits in humans. The studies were primarily conducted in animal models, and further research is needed to determine the optimal dosages and confirm these effects in human populations. What is worth mentioning is that currently metformin is in a process to be established as one of the AD medications [73].

Outdoor Activity and Nature Contact

Engagement in pro-nature physical activities and outdoor exercise has demonstrated significant potential in enhancing cognitive function and mitigating psychological stress through their influence on the MGBA. Engaging in these activities leads to a greater variety of gut microbiota, boosts the presence of helpful bacteria, and encourages the formation of SCFAs, which are crucial for preserving gut balance [74,75]. Outdoor activities and interactions with the natural environment can positively affect the microbiome and its metabolites. Studies have indicated that being in environments abundant in biodiversity can boost the levels of Firmicutes and Akkermansia bacteria, which in turn affect the hypothalamic-pituitary-adrenal (HPA) axis, brain-derived neurotrophic factor (BDNF), and serotonin pathways [75]. Engaging with green spaces has been associated with a decreased relative abundance of Bacteroidetes, Bacteroides, and Anaerostipes, while showing an increased relative abundance of Lachnospiraceae and Ruminococcaceae [76]. The proposed mechanism underlying these microbiota changes involves the reduction of perceived psychological stress, as evidenced by lower scores on the Perceived Stress Scale (PSS) in pediatric populations [76]. Additionally, engaging in moderate-to-vigorous physical activity (MVPA) promotes the breakdown and absorption of gut-derived anti-inflammatory and immunomodulatory metabolites into the bloodstream [74]. The integration of a flavonoid-rich diet with regular aerobic exercise, particularly in natural environments, may enhance cognitive benefits and mitigate cognitive decline in aging populations. This effect is likely mediated by the gut microbiome through mechanisms such as increased microbial diversity, enhanced SCFAs production, and improved gut barrier integrity [74,77]. Nonetheless, further research-including mechanistic animal studies and well-designed human clinical trials-is essential to comprehensively elucidate the relationship between outdoor activity, nature contact, and cognitive function. Currently, evidence is limited to preliminary findings from small-scale studies, and no definitive causal relationship has been established. However, this emerging field offers promising insights into the intricate interplay of the GBA, highlighting the potential role of environmental exposures in modulating gut microbiota and influencing cognitive health.

## Conclusions

Growing evidence highlights the gut microbiota and its metabolites as promising targets for early intervention in cognitive impairment. Modifying the gut ecosystem through diet and probiotics may offer practical and effective strategies for maintaining cognitive function, particularly in the early stages of decline. Among these, adherence to the Mediterranean or MIND diets is strongly supported by clinical data and can be readily recommended to patients. In parallel, probiotic supplementation-particularly with strains of *Lactobacillus *and *Bifidobacterium* - has shown modest but consistent cognitive benefits in both animal models and human studies, and may be considered as adjunctive therapy in individuals with MCI. Additionally, metformin, widely used in metabolic disorders, may offer cognitive benefits through its impact on gut microbiota composition and systemic inflammation. While other interventions such as prebiotics, synbiotics, or FMT remain investigational, a multimodal approach combining dietary changes, probiotics, and metabolic control represents a viable clinical strategy. Clinicians should consider these evidence-based interventions as part of early, proactive management of cognitive decline.
